# Primary myoepithelial carcinoma of the lung: a rare entity treated with parenchymal sparing resection

**DOI:** 10.1186/1749-8090-6-27

**Published:** 2011-03-08

**Authors:** Inderpal S Sarkaria, Deborah DeLair, William D Travis, Raja M Flores

**Affiliations:** 1 Department of Surgery, Thoracic Service, Memorial Sloan-Kettering Cancer Center, 1275 York Avenue, New York, NY, 10065, USA; 2 Department of Pathology, Memorial Sloan-Kettering Cancer Center, 1275 York Avenue, New York, NY, 10065, USA; 3 Division of Thoracic Surgery, Mount Sinai Medical Center, 1190 Fifth Avenue, PO Box 1028, New York, NY, 10029, USA

## Abstract

Primary lung myoepithelial carcinomas are rare neoplasms arising from the salivary glands of the respiratory epithelium. Given the rare occurrences and reports of these tumors, appropriate recommendations for resection are difficult to formulate. Although classified as low-grade neoplasms, these tumors have a significant rate of recurrence and distant metastasis.

## Introduction

Primary salivary gland-type tumors of the lung are rare and include mucoepidermoid carcinoma, adenoid cystic carcinoma, acinic cell carcinoma, oncocytoma, epithelial-myoepithelial carcinoma, benign myoepithelioma, and mixed tumors of both benign and malignant nature [1-16]. Primary pulmonary myoepithelial carcinomas are exceedingly rare, with only five known prior cases reported in the English literature to date [17-20]. We report a case of primary myoepithelial carcinoma of the lung and a review of the literature.

## Case Report

**Figure 1:**
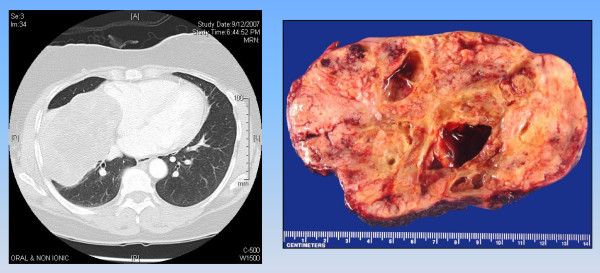
**Radiographic and gross pathologic appearance of primary pulmonary myoepithelial carcinoma**. **A**. Computed tomography of right-sided chest mass with compression of right atrium. **B.** Gross pathologic appearance of right visceral pleural mass.

A 63-year-old woman presented to the clinic with a history of increasing pulmonary congestion, difficulty expectorating, and low-grade hemoptysis. Her past medical history was significant for diet-controlled insulin-dependent diabetes mellitus. There was no history of cigarette or other tobacco use. Her mother had died of breast cancer in her fifties. A chest radiograph showed a large mass in the right lower lung fields, and computed tomography scanning revealed a 12.4 × 8.3 centimeter smoothly circumscribed heterogeneous mass arising in the right minor fissure and impinging upon the right atrium (Figure 1A). A fine needle aspiration was performed which yielded a diagnosis initially suspicious for schwannoma, as reviewed at an outside institution. At the time of thoracotomy, dense adhesions were dissected free of the upper and middle lobes with no evidence of tumor invasion, and the mass was resected en-bloc with pericardium and a wedge resection of the lower lobe. An additional separate lower lobe nodule was also excised and presumed to be a metastatic focus of tumor. The patient had an uneventful recovery and was discharged home on post-operative day number three.

The patient developed a biopsy proven solitary liver/diaphragmatic metastasis diagnosed on routine follow-up at 36 months post-resection.

## Pathologic Findings

Gross pathologic examination revealed the mass to be a 13 × 8 × 8 centimeter lower lobe carcinoma of myoepithelial origin involving the visceral pleura (Figure 1B).

Histologically, the mass was consistent with a malignant myoepithelial neoplasm with a fibrous capsule and 20% necrosis. The mass was thought to arise from the right lower lobe and involved the visceral pleura. The margin was focally within 0.1 cm of the tumor, but otherwise grossly free of invasion. The separate nodule was 1 cm in size and histologically similar to the primary tumor.

Immunohistochemical stains were performed and were focally positive for AE1/AE3, Bcl2, Cam5.2, S100p, GFAP, 4A4, SMA, and CD99. Stains for EMA, desmin, CD34, calponin, FLI1, myogenin, and synaptophysin were negative.

## Discussion

Myoepithelial carcinomas primarily arise from the salivary glands, the parotid, or the breast [21]. Rarely, they may arise in soft tissues, most often in the lower and upper limbs, occurring equally in men and women [22]. These soft-tissue tumors, distinguished from benign myoepitheliomas by their moderate or severe cytologic atypia or invasive growth pattern, recur locally in 42% of patients and metastasize to distant sites in 32%.

Primary myoepithelial carcinomas of the lung are exceedingly rare, with only 6 patients reported in the English literature, including the present report [17-20]. Clinical data from these reports are summarized in Table 1. Very limited information was available for one patient [20]. Most patients were male, in their fifth or sixth decade of life at diagnosis, and smoked tobacco. Most patients presented with primarily endobronchial disease, had preoperative biopsies of their tumors, and underwent anatomic resections of these malignancies. There were no local recurrences. Almost all patients had metastatic disease, either synchronous at time of resection, or presenting as metachronous recurrence. Follow-up was less than two years for all but one patient, who died of metastatic disease to the liver five years postoperatively. One other patient died at fourteen months from synchronous metastatic adenocarcinoma. One patient was alive with disease, and two others were free of disease at relatively short follow-up intervals.

**Table 1:** Clinical characteristics of reported cases of myoepithelial carcinoma of the lung.

AUTHOR	Age/Sex	Smoking	Location	Pre-op Bx Diagnosis	Size (cm)	Resection Type	Local Recurrence	Metastasis	Outcome
Present case	63F	No	RLL, Pleural & parenchymal	Low grade spindle cell neoplasm	13.0	Wedge/Local excision	No	Yes, same lobe/pleura at time of resection, liver at 36 months	AWD at 36 months

Masuya 2005	48M	Yes	LLL, Parenchymal & Endobronchial	Sarcoma, sarcomatoid carcinoma, carcinosarcoma	1.5	Lobectomy	No	No	NED 15 months

Miura 2000	46M	NA	Right hilum, Right Main Stem, Endobronchial	No atypical cells	6.5	Pneumonectomy	No	Yes, LLL	AWD at 7 months

Higashiyama	58M	Yes	RUL, Endobronchial	NA	3.8	Sleeve bilobectomy (RUL/RML)	No	Yes, soft tissue left arm and hip	DOC at 14 months (metastatic synchronous adenocarcinoma)

Higashiyama 1998	58M	Yes	LUL, Endobronchial	Squamous cell carcinoma	6.0	Sleeve lobectomy (LUL)	No	Yes, liver	DOD, 60 months

Sekine 1998	NA	NA	NA	NA	NA	NA	NA	Yes	NA

RLL - right lower lobe; LLL - left lower lobe; RUL - right upper lobe; LUL - left upper lobe; RML - right middle lobe; Bx - biopsy; NED - no evidence of disease; AWD - alive with disease; DOC - dead of other causes; DOD - dead of disease

Given the available reported data, there are a number of unique characteristics of the current case when compared to the previous five. This case represents the eldest and only female patient, as well as the only known never-smoker. This patient's tumor is also the greatest in size within this series, more than doubling the previous known largest of these tumors. All other patients in the series presented with a major component of endobronchial disease, whereas the current tumor was primarily pleural/parenchymal based. Finally, the current case represents the only patient treated with a limited sub-anatomic resection.

Given the relatively high rates of recurrence, low-grade malignant status, and the propensity for recurrence at distant sites, it is reasonable to consider limited sub-anatomic, parenchymal sparing resections for these patients, especially if pneumonectomy is contemplated. While this may not be adequate for endobronchial lesions involving major pulmonary segments, it is feasible for lesions presenting with primarily parenchymal or pleural based disease, as in the current case. Given the rarity of these tumors, recommendations regarding chemotherapy or radiation, either pre- or postoperatively, are difficult to formulate.

## Consent

Written informed consent was obtained from the patient for publication of this case report and accompanying images. A copy of the written consent is available for review by the Editor-in-Chief of this journal.

## Competing interests

The authors declare that they have no competing interests.

## Authors' contributions

ISS performed study design, data collection, and primary manuscript preparation and revision. DD performed gross, histological, and molecular pathology review and interpretation. WDS performed study design, pathology review, and manuscript preparation and revision. RMF performed study inception, study design, study oversight, and manuscript review and revision. All authors have read and approved the final manuscript.
